# Therapy-based expert system on function and postural stability after anterior cruciate ligament reconstruction: a pilot study

**DOI:** 10.1186/s12891-023-06735-w

**Published:** 2023-07-29

**Authors:** Anoshirvan Kazemnejad, Abbas Asosheh, Azar Moezy, Arezoo Abasi

**Affiliations:** 1 Department of Biostatistics, Faculty of Medical Sciences, Tarbiat Modares University, Tehran, Iran; 2 Department of Medical Informatics, Faculty of Medical Sciences, Tarbiat Modares University, Tehran, Iran; 3 Department of Exercise and Sports Medicine, School of Medicine, Hazrat-E Rasool General Hospital, Iran University of Medical Sciences, Tehran, Iran

**Keywords:** Anterior Cruciate Ligament Reconstruction, Expert System, Wii Fit, Rehabilitation

## Abstract

**Purpose:**

Wii Fit exergames have been less commonly used for the rehabilitation of athletes after Anterior Cruciate Ligament Reconstruction (ACLR). This study aims to investigate the effects of an expert system using Wii Fit exergames compared to conventional rehabilitation following ACLR. A forward-chaining rule-based expert system was developed which proposed a rehabilitation program that included the number and type of exercise in terms of difficulty and ease and the duration of each exercise in a progressive manner according to the patient's physical condition.

**Materials and methods:**

Twenty eligible athletes aged 20–30 who underwent ACLR were enrolled in this study and randomly assigned to two groups; and received 12 sessions of either Wii Fit exergames as Wii group (*n* = 10) or conventional rehabilitation as CL group (*n* = 10).

**Results:**

The main outcomes consisted of pain (Visual Analogue Scale (VAS)), knee effusion, knee flexion range (KFR), thigh girth (TG), single-leg hop for distance (SLHD), and for time (SLHT), static and dynamic balance tests. Both groups had considerable improvement in all outcomes, also there were significantly differences between Wii and CL groups as follows; VAS (*P* < 0.001), knee effusion (*P* < 0.001), TG (*P* = 0.001), KFR (*P* = 0.012), static balance in stable position (*P* < 0.001) and in unstable position (*P* = 0.001), dynamic balance in the anterior (*P* < 0.001), posteromedial (*P* < 0.001), posterolateral (*P* = 0.004) directions, symmetry index of SLHD (*P* < 0.001) and symmetry index of SLHT (*P* = 0.013).

**Conclusions:**

The findings showed that using Wii Fit exergames in post-ACLR patients reduced pain and effusion while also improving function and balance significantly. Iranian Registry of Clinical Trials registration number is IRCT20191013045090N1, and the registration date is 03-03-2020.

## Introduction

Anterior Cruciate Ligament (ACL) plays a crucial role in proper knee function and stability [1]. The ACL injury rates are increasing significantly during recent years [2]. ACL injuries are associated with pain, effusion, loss of motion, muscle atrophy, proprioceptive impairment, and postural instabilities [3]. Therefore, most patients prefer to undergo ACLR to achieve normal function [4]. One of the important goals of ACL rehabilitation is to further encourage the patient to participate in rehabilitation programs to improve strength, range of motion (ROM), postural stability, agility, and coordination.

Wii Fit™ is an exergaming video game released by Nintendo Wii™(Nintendo Co Ltd, Kyoto, Japan) with 48 exergames [5]. Exergaming (exercises utilizing video game technology) is a fairly new approach to improve physical activity and stability by combining exercise with gaming. The charm of exergames will detach the patient's mind from the repetitive and tedious nature of rehabilitative tasks and draw them to the enjoyable aspects of exergames [6, 7].

The Nintendo Wii Balance Board (NWBB) is a unique accessory for the Wii console designed to train and also evaluate the center of balance [8, 9]. Clark and Howells and their colleague have used it to assess postural control and weight-bearing asymmetry after ACLR, but not for the rehabilitation [10–12]. Immediate feedback, intensive work-based therapy, and the potential to increase patient motivation are some of the most important capabilities of the NWBB, providing an attractive tool for ACLR rehabilitating [13, 14].

In the review of the use of NWBB in ACL rehabilitation, Karakoc et al. found no significant differences in pain, functionality, and balance between Wii Fit and control groups [15]. Similarly, Baltaci et al. did not report significant differences after Hamstring ACLR rehabilitation between Wii Fit and conventional group in terms of isokinetic knee strength, dynamic balance, and functional squat tests [16]. According to these studies the Wii Fit was considered as effective as conventional methods. In these studies, all participants used the same Wii Fit exergames program without considering each patient's specific condition.

Expert System (ES) has recently been introduced in rehabilitation with the capacity to replace the specialist. ES is used to solve problems with explicit knowledge but without any special algorithm [17–19]. Increasing efficiency, reducing costs, saving time, reducing workload, clarifying reasoning, and responding regularly, consistently, and non-emotionally at all times are the most outstanding benefits of ES [20]. A rule-based ES comprises information collected from a human expert and expresses that information using rules like IF–THEN. The rules can then be used to perform operations on data in order to infer the correct conclusion [21]. There are a lot of applications of rule-based systems in ES in medicine [22, 23]. ES, a relatively untouched domain of physiotherapy, consists of an inference engine, knowledge base, working memory, an explanation center, and User Interface (UI) [24]. To the best of our knowledge, few studies have been performed to evaluate the effectiveness of the NWBB in ACL rehabilitation without designing ES [16].

This research has focused on developing an ES for providing a rehabilitation program using NWBB exergames compared to the conventional rehabilitation on balance and physical function of ACL-reconstructed athletes.

In summary, this study aims to develop an expert system (ES) for ACL rehabilitation using the Nintendo Wii Balance Board (NWBB) exergames and compare its effectiveness with conventional rehabilitation methods on ACL-reconstructed athletes' balance and physical function. The current literature suggests that the use of NWBB without an ES may not result in significant differences in rehabilitation outcomes compared to conventional methods. This study's original and innovative aspect is the development of an ES for ACL rehabilitation using NWBB exergames, which has not been extensively studied in physiotherapy. The study will enroll 20 ACL-reconstructed athletes, randomly assigned into two groups, one using the ES-based Wii Fit exergames program and the other using conventional rehabilitation. The study's outcomes will include pain, knee effusion, knee flexion range, thigh circumference, and single-leg hop for distance and time, as well as static and dynamic balance tests. The results of this study may contribute to improving ACL rehabilitation outcomes and provide a new approach to physiotherapy rehabilitation.

## Material and methods

The study was designed as a pilot double-blinded randomized controlled trial carried out on 20 male athletes who underwent unilateral arthroscopic ACLR surgery with bone-patellar tendon-bone graft between March 2020 and March 2021. The Research Ethics Committee of Tarbiat Modares University approved the study (IR.MODARES.REC.1398.124) and all patients provided written informed consent. The study was carried out based on the latest version of the Declaration of Helsinki. Our study was also approved as a pilot randomized controlled trial study in the Iranian Registry of Clinical Trials with the ID of IRCT20191013045090N1 on 03-03-2020.

Also, the patients signed a written Persian consent form and announced their agreement to participate in the study and for publication of the results with respect to the confidentiality of personal data.

### Participants

All participants were primarily checked for eligibility criteria before being referred to the physical therapy clinic. The inclusion criteria were as follows: (1) patient with ACLR surgery at least three months ago; (2) aged between 20 to 30 years; (3) BMI ≤ 30; (4) active ROM on reconstructed knee ≤ 90°; (5) normal mental state. The exclusion criteria were (1)previous or concomitant injury or surgery on the relevant knee and other joints; (2) history of lower extremity fractures surgery or traumatic injuries within the last six months; (3) neuromuscular disease; (4) bone implants; and (5) history of any diseases that restrict activities and any other conditions affecting the study; (6) usage of opioid analgesics or systemic corticosteroids within the last four weeks; (7) inability to do exercise; (8) any damages during the study; (9) unwillingness to participate in the study; (10) engagement in out-of-schedule exercises; (11)receiving any treatment other than the programs prescribed in this research; and (12) incomplete assessment/treatment programs. Before enrollment, the patients who were included in the study signed university-approved written informed consent forms and completed demographic datasheets. All participants were permitted to withdraw at any time if they did not wish to pursue the study. The sample size was calculated 10 in each group by considering type one (α) and type two errors (ß) of 0.05 and 0.20 (power = 80%), respectively, to detect the effect size of two scores in pain improvement scale (VAS).

### Randomized allocation

**Fig. 1:**
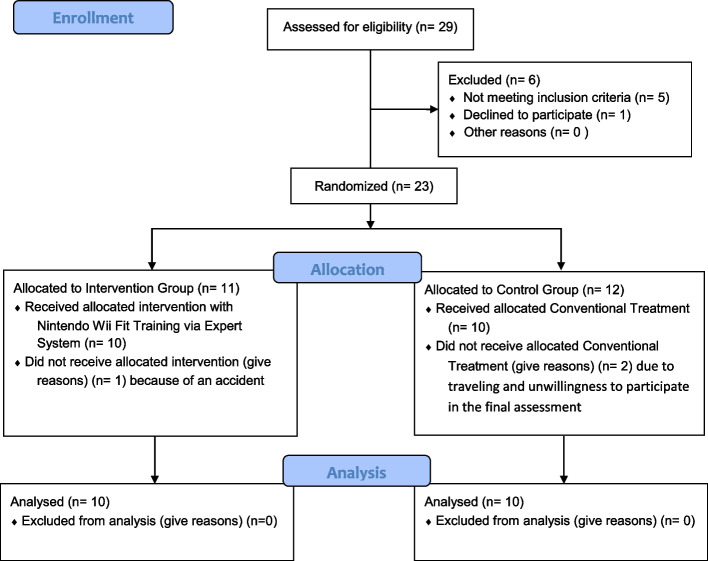
CONSORT Flow diagram of the participants

Initially, 29 patients were enrolled to study; five of them did not fulfill the inclusion criteria. The eligible participants were 23 patients who were randomly allocated into two groups: (1) Nintendo Wii Fit system (named Wii group) as an intervention group, and their exercise program was given by a proposed ES. (2) The control group received a conventional ACL rehabilitation program (named CL group). Three athletes were also excluded in the next stage (Fig. 1). The patients who met the inclusion criteria, randomly assigned to one of the groups using a block randomization process.

The patients who met the inclusion criteria were randomly assigned to one of the groups in a 1:1 ratio using a computer-generated random allocation sequence using a stratified block randomization process. Patients were blinded to treatment. Also, assessors were blinded to the allocated group of patients.

### Assessment procedures

The patient underwent a baseline assessment including a demographic checklist, Visual Analog Scale (VAS) for pain, Knee flexion Range (KFR), Thigh Girth (TG), knee effusion, Balance Error Scoring System (BESS), Modified STAR Excursion Balance Test (MSEBT) and two functional tests of Single-Leg Hop for Distance (SLHD) and Time (SLHT). The outcomes were measured in two intervals; at the baseline or pre-intervention and at the 4th week or post-intervention in the physical therapy clinic by assessors.

### Proposed expert system

**Fig. 2:**
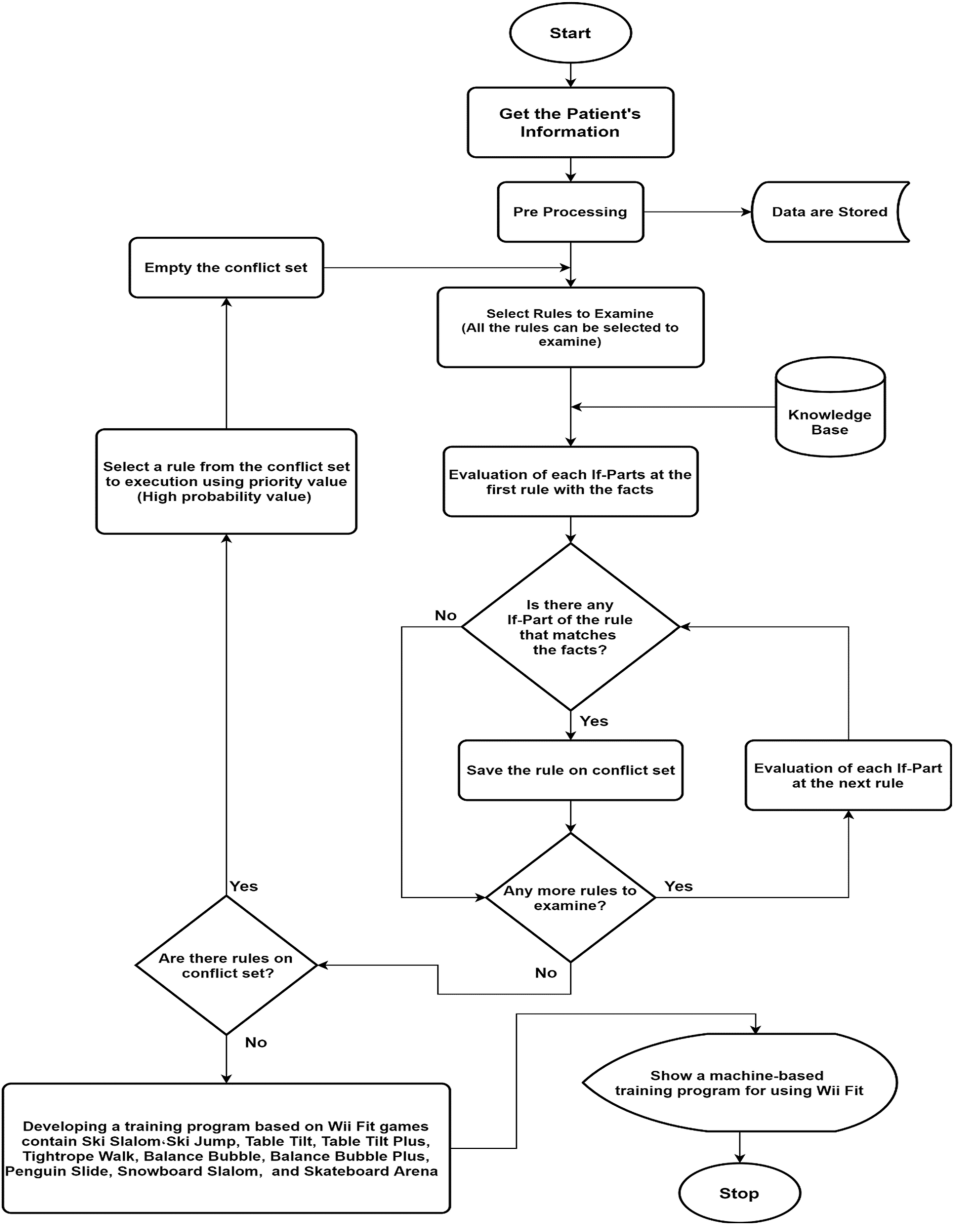
The flowchart of the proposed ES

The proposed ES (Fig. 2) has been developed to evaluate the patients’ conditions and present an appropriate rehabilitation program according to the assessment findings, analyzing the outcomes, and tracking the patient's progress.

The main sources of knowledge for rehabilitation after ACLR were obtained from sports medicine and physiotherapist through several interviews, debates, and surveys of the Wii Fit exergames. According to the experts’ responses, the most important assessment variables for optimal rehabilitation consisted of knee effusion, TG, KFR, BESS, and MSEBT used as ES inputs.

After the exergames verifying, ten exercises were chosen including; Ski Slalom, Ski Jump, Table Tilt, Table Tilt Plus, Tightrope Walk, Balance Bubble, Balance Bubble Plus, Penguin Slide, Snowboard Slalom, and Skateboard Arena with three levels of difficulty; beginner, advanced, and expert.

After collecting and analyzing data of the patients by ES, preliminary calculations were carried out to determine the differences of effusion, TG, and KFR between healthy and reconstructed knees.

The BESS scores were checked, the differences of Double-Leg (DL), Single-Leg (SL), and Tandem (T) from stable and unstable positions were obtained and summed up, and finally, their means were calculated (Eq. 1).

The reach distances of the MSEBT test in three directions and their mean were calculated (Eq. 2).

The ES forward-chaining strategy is an inferential method that begins with a set of known facts and generates new facts using rules that are applied to the facts. This process continues until a rule can no longer be used in the inference process [25]. The inference engine prioritizes the satisfied rules and executes the rule with the highest priority for planning ACL rehabilitation.

1 $$BESS= {}^{\left(\left|Stabl{e}_{DL}- Unstabl{e}_{DL}\right|+ \left|Stabl{e}_{SL}- Unstabl{e}_{SL}\right|+ \left|Stabl{e}_{T}-Unstabl{e}_{T}\right| \right)}\!\left/ \!{}_{3}\right.$$

2 $$MSEBT={}^{(Anterior + PosteroMedial +PosteroLateral)*100}\!\left/ \!{}_{(3*Lower\_Limb\_Length)}\right.$$

Designed rules will select Wii Fit exergames according to the patient's condition. The output of the ES is a training program based on ten selected games with its level of difficulty and duration. The rules are planned to make it possible to exercises easier form while the patient is in a poor situation. The duration and difficulty of the rehabilitation program have gradually grown to ensure that treatment progresses.

The UI is intended to communicate between the user and the ES and compare the patient's condition in different sessions using graphical charts. Through this reporting system, the patient can assess his condition during the rehabilitation. Another part of the UI was the scoring system to evaluate the progress of the patient's rehabilitation during 12 sessions.

According to the experts' opinions, the patients were labeled in five categories; deterioration, unchanged, low, moderate, and excellent based on their improvement by the scoring system.

The ES was developed by Python version 3.6 and graphical UI by Tkinter-toolbox. The Microsoft SQL Server was used to store the data. After preliminary evaluation of the proposed ES by an experienced orthopedic surgeon and a sports physician, they expressed their satisfaction with its performance and ensuring the system’s rules validity.

### Interventions

All participants participated in 12 supervised 90-min exercise sessions; three times a week consisting of 15-min warm-up plus lower limb stretches, 60-min of an ES-based protocol based on the Wii Fit or conventional protocol, 15-min lower limb stretches, and a cool-down period. The patients had the same warm-up schedule in both groups that consisted of a 10-min jogging warm-up program on a treadmill (Technogym, Italy) with 5-min gentle stretches of quadriceps, hamstring, hip adductor, and calf (3–5 stretches/muscle group; hold 30-s). Prior to the exercise-therapy, the patients in the Wii group were asked to read the instructions and the safety guidelines of the console. In the first, fourth, seventh, and tenth sessions, the ES progressed the Wii Fit program for each participant after analyzing their outcomes. The exercise programs for patients in the CL group included the following:

- Static exercises for quadriceps, hamstring, gluteal muscles, and SLR.
- Closed chain exercises (wall sit, mini squat, and leg press).
- Hip muscles strengthening exercises.
- Proprioceptive exercises.
- Walking and jogging on a treadmill.

Before the exercise therapy, all participants received High TENS (Combined BTL-4825S Topline, UK) for 20-min. Also, all participants were asked to avoid taking pain killer one week before and during the intervention to unify the participants' medications. Acetaminophen was only prescribed with a maximum dose of 2g daily if they experienced pain.

### Statistical analysis

Statistical analyses were performed using SPSS version 25 (SPSS Inc., Chicago, IL, USA). The results were reported as mean ± Standard Deviation (SD) for quantitative variables and were summarized for categorical variables by frequency (percentage). Kolmogorov–Smirnov test was used to investigate the normal distribution of quantitative data. A paired-sample t-test was used to determine the within-group differences. An independent sample t-test was used to compare the mean of the two groups when they had a normal distribution. Fisher's exact test was used to compare the ratios in the two groups. The significance level was set at *P* < 0.05.

Ten patients with the same reconstructive surgery had repeated measurements seven days apart in a pilot study and the Intra-class Correlation Coefficients (ICC) for BESS, MSEBT, SLHD, and SLHT were 0.92, 0.95, 0.94, and 0.97, respectively, with a 95% level of confidence.

## Results

CONSORT diagram detailing the flow of participants through each stage of the trial can be seen in Fig. 1. Out of 29 individuals following ACLR, 23 were qualified to be included in the study. Within training sessions, three patients left the study including two patients in the CL group (due to traveling and unwillingness to participate in the final assessment.), and one in the Wii group (because of an accident); and the rest completed all training and assessment sessions.

No significant differences were observed between Wii and CL groups for the demographic variables (Table 1) using an independent sample t-test, indicating the groups were well matched. In both groups, 90% of the participants were right-dominant, seven in the Wii group, and eight in the CL group have had left-knee ACL reconstruction. The exergames were pleasant and non-fatiguing for all patients in the Wii group.

**Table 1:** Comparison of the mean and standard deviation of demographic and clinical variables in WII and CL groups before the intervention

*Demographic Variables*	*CL Group (n* = *10)* *Mean* ± *SD*	*Wii Group (n* = *10)* *Mean* ± *SD*	*P-value*
*Age (years)*	*24.4* ± *3.16*	*24.7* ± *4.24*	*0.860*
*Weight (kg)*	*79.4* ± *12.85*	*85.4* ± *13.6*	*0.324*
*Height (meters)*	*177.6* ± *6.19*	*181.2* ± *4.84*	*0.171*
*Body mass index (Kg / m2)*	*25.1* ± *3.31*	*25.9* ± *3.55*	*0.583*
*Lower limb length (meters)*	*96.2* ± *4.89*	*98.7* ± *2.65*	*0.178*
*Time from surgery to the pre-intervention assessment of the study (months)*	*3.8* ± *0.63*	*3.8* ± *0.71*	*1*

At the beginning of the analysis, the independent sample t-test indicated no significant differences in the clinical variables between the two groups (Table 2).

**Table 2:** Changes in variables within and between the Wii and CL groups pre- and post-intervention

	**Wii Group (*n* = 10)** **Mean ± SD**	**CL Group (*n* = 10)** **Mean ± SD**	***P-value***
*VAS (0–10)*			
Pre-intervention	3.60 ± 0.96	3.45 ± 0.99	*0.128*^*b*^
Post-intervention	1.20 ± 0.63	2.39 ± 0.66	*0.013*^*a*^
*P-value*	< 0.001^*c*^	< 0.001^*c*^	
*Knee Effusion (cm)*			
Pre-intervention	44.20 ± 3.25	41.25 ± 3.66	*0.073*^*b*^
Post-intervention	42.05 ± 2.82	40.45 ± 3.55	*0.280*^*e*^
*P-value*	< 0.001^*c*^	< 0.001^*c*^	
*TG (cm)*			
Pre-intervention	48.70 ± 6.16	45.90 ± 5.44	0.296^*b*^
Post-intervention	51.05 ± 6.05	46.95 ± 5.26	0.124^*e*^
*P-value*	< 0.001^*c*^	< 0.001^*c*^	
*KFR (degree)*			
Pre-intervention	115.00 ± 9.82	117.00 ± 8.58	0.634^*b*^
Post-intervention	127.00 ± 6.01	122.10 ± 7.34	0.023^*e*^
*P-value*	< 0.001^*c*^	0.001^*c*^	
*BESS in stable condition (number of errors)*			
Pre-intervention	15.00 ± 5.73	17.02 ± 3.25	0.306^*b*^
Post-intervention	8.90 ± 4.45	15.65 ± 1.96	0.014^*a*^
*P-value*	< 0.001^*c*^	0.781^*d*^	
*BESS in unstable condition (number of errors)*			
Pre-intervention	26.80 ± 4.56	26.00 ± 2.98	0.648^*b*^
Post-intervention	15.70 ± 4.05	24.60 ± 2.41	0.048^*a*^
*P-value*	< 0.001^*c*^	0.231^*d*^	
*MSEBT Anterior Reach Index (%)*			
Pre-intervention	125.16 ± 15.31	131.98 ± 18.91	0.342^*b*^
Post-intervention	134.95 ± 12.54	132.62 ± 19.43	0.005^*a*^
*P-value*	< 0.001^*c*^	0.091^*d*^	
*MSEBT Posteromedial Reach Index (%)*			
Pre-intervention	125.09 ± 20.22	131.59 ± 20.06	0.480^*b*^
Post-intervention	134.89 ± 20.12	132.57 ± 20.51	0.012^*a*^
*P-value*	< 0.001^*c*^	0.561^*d*^	
*MSEBT Posterolateral Reach Index (%)*			
Pre-intervention	131.44 ± 10.41	131.92 ± 12.80	0.934^*b*^
Post-intervention	141.45 ± 10.22	132.86 ± 14.89	0.042^*a*^
*P-value*	< 0.001^*c*^	0.301^*d*^	
*SLHD symmetry index (%)*			
Pre-intervention	80.78 ± 8.12	86.47 ± 8.91	0.153^*b*^
Post-intervention	88.14 ± 9.64	88.13 ± 9.27	0. 017^*a*^
*P-value*	< 0.001^*c*^	0.009^*c*^	
*SLHT symmetry index (%)*			
Pre-intervention	140.67 ± 26.21	137.10 ± 29.20	0.777^*b*^
Post-intervention	127.05 ± 22.48	134.68 ± 24.75	0.024^*a*^
*P-value*	0.003^*c*^	0.339^*d*^	

^a ^Significant difference between groups (independent sample t-test) post-intervention

^b ^Non-significant difference in the baseline between groups (independent sample t-test) (*P* > 0.05)

^c ^Significant difference within-group (paired t-test)

^d ^Non-significant difference within-group (paired t-test) (*P* > 0.05)

^e ^Non-significant difference between groups (independent sample t-test) post-intervention

Differences within the group were analyzed using the paired t-test. Significant differences were found in all variables in the Wii group; while VAS, Knee effusion, TG, and KFR revealed significant differences in the CL group in within-group comparison (Table 2).

Between-group differences were analyzed using an independent sample t-test and significant improvements were observed in all studied variables (*P* < 0.05) after the interventions, except for knee effusion, and TG (Table 2). In addition, the rate of progression of all variables is presented in Table 3 for further comparison of the two groups. There were statistically significant differences in the rate of progression of all the findings between the groups.

**Table 3:** Progression rate of the variables in the Wii and CL groups by the independent sample t-test

	***Wii Group (n* = *10)*** ***Mean* ± *SD***	***CL Group (n* = *10)*** ***Mean* ± *SD***	***P-value***
*VAS*	*66.50* ± *15.76*	*29.83* ± *17.54*	< 0.001*
*Knee Effusion*	*4.81* ± *1.57*	*1.93* ± *0.58*	< 0.001*
*TG*	*4.93* ± *1.65*	*2.35* ± *1.08*	0.001*
*KFR*	*10.87* ± *5.36*	*4.48* ± *2.87*	0.012*
*BESS in stable condition*	*41.71* ± *12.57*	*23.12* ± *5.78*	< 0.001*
*BESS in unstable condition*	*42.12* ± *8.21*	*28.30* ± *6.80*	0.001*
*MSEBT Anterior Reach Index*	*8.19* ± *4.04*	*1.44* ± *0.81*	< 0.001*
*MSEBT posteromedial Reach Index*	*8.09* ± *3.83*	*2.26* ± *0.77*	< 0.001*
*MSEBT posterolateral Reach Index*	*7.72* ± *3.12*	*3.78* ± *2.19*	0.004*
*SLHD symmetry index*	*9.04* ± *2.86*	*2.91* ± *0.69*	< 0.001*
*SLHT symmetry index*	*14.62* ± *4.37*	*3.13* ± *0.19*	0.013*

^* ^Significant Difference

## Discussion

Due to the growing number of people with ACL injuries and their urgent need for post-operative rehabilitation, numerous clinicians are seeking to develop new protocols to accelerate the rehabilitation process. With its board and exergames, Wii Fit could offer enjoyable training and accurate feedback to improve the outcomes. A new exergame protocol based on the expert system has been proposed to accelerate the ACLR rehabilitation process in the study.

In the proposed ES, after entering the data and initial calculations, a proper rehabilitation program was presented in the format of ten Wii Fit exercises with their repetition, duration of each exercise, and difficulty level. Reducing waste of time is one of its most important advantage over conventional approaches. The patient could receive a training regimen from the ES without the need for constant attendance of therapists. With the ES increasing accessibility, the insights of the best experts are provided to the user anywhere and anytime through the computer. In the present research, the effectiveness of ES-based Wii Fit protocols on pain, knee effusion, TG, KFR, physical function, balances compared to the conventional rehabilitation outcomes in the patients following ACLR.

Pain is one of the annoying complications after ACL reconstructive surgery that might be endured for months. Persistent pain often leads to neuromuscular inhibition, muscle atrophy, and reduction of knee ROM [26, 27]. According to this study, both exercise programs were successful in decreasing pain. However, the pain reduction was significantly higher in the Wii group than the CL group, which may be attributed to increased muscle strength due to the appropriate selection of specific exercises by ES. The precise reasons for reducing knee pain after exercise-therapy remain unclear; different analgesic processes tend to be involved, such as endogenous opioid and non-opioid processes. There is also evidence that Exercise-Induced Hypoalgesia can effectively increase the pain threshold and enhance beta-endorphin release and other analgesics in the central nervous system following exercise [28, 29]. Contrary to our results, Karakoc et al. did not observe any significant difference in pain between the two groups [15]. This may be due to the inconsistency of the exercise program with the patients’ conditions. Also, Wibelinger et al. [28] found no significant difference in pain in patients with knee osteoarthritis using the Wii system. The reason for this discrepancy might be attributed to the elderly patients with knee osteoarthritis studied by Wibelinger et al., while the participants in our study were young athletes who underwent ACLR. In either case, the results of this research demonstrate that exergame with a Wii Fit is as effective as traditional exercise-therapy in reducing pain.

Joint effusion is seen in most patients after ACLR which causes several complications loss of motion, neuromuscular inhibition, muscle atrophy, decreasing postural stability, and abnormal gait [27]. Our finding has revealed no significant difference between the groups post-intervention in joint effusion. It seems that exergame reduces joint swelling indirectly by activating the muscle pump, similar to conventional exercises which improve intravenous and lymphatic drainage in the lower extremities. However, there was a significant difference in the progression rate between the groups. The attractiveness and the type of exergame, which motivates the person to work longer may be the reason for this progression. Unfortunately, knee effusion has less been evaluated in other studies.

The measurement of TG is commonly used to assess thigh muscle bulk atrophy. According to our findings, TG demonstrated no significant difference between the two groups similar to Karakoc et al. [15]. Nevertheless, in terms of progress rate, we observed a significant difference between the groups, which may be due to the better selection of exercise protocol by ES.

Loss of mobility is one of the undesirables of ACL reconstructive surgery caused by pain, surgery, effusion, inactivity, periarticular tissue fibrosis, adaptive shortening of muscles, and so on [3]. There was a significant difference between Wii and CL groups in terms of increasing KFR. Also, the progression rate of KFR in the Wii group was remarkable. Similarly, Puh et al. [29] have reported that the Wii Fit exercises have dramatically increased knee flexion on a case after posterior cruciate ligament (PCL) reconstruction. It seems more passion for exercise with Wii Fit is the possible reason for the KFR improvement.

Hopping tests are among the most important functional assessments after ACLR with high validity and reproducibility in healthy and reconstructed knees [3, 30]. These functional tests are revealed significant differences between the groups based on our results. Furthermore, there were significant differences in the symmetry indices of functional tests and their progression rate indicating an improvement in the physical function of the Wii group. Contrary to our findings, Baltaci et al. [16] and also, Karakoc et al. [15] have not obtained any significant differences in the function between the Wii Fit and CL groups. The variations in surgical techniques, patients’ fitness, or exercise programs may be the cause for the different outcomes between ours and these studies. It should also be noted that the use of ES and the optimal choice of exercises to improve patients' function might be the key source for these discrepancies. Similar to the results of the present study, Vojciechowski et al. [31] demonstrated great progress in the results of functional tests in the elder women who were at risk for falls and bone fractures after exercising with Wii Fit. Of course, the results of our study are not comparable to the findings of Vojciechowski et al., since our samples were young male athletes, while Vojciechowski et al. has studied old women. However, in general, it seems that training with the Wii Fit system can play an effective role in improving daily activity and physical performance.

ACL plays a vital role as a sensory organ supplying proprioceptive data that initiates a protective muscular reflex. Reconstruction of ACL decreases its proprioceptive ability and impairs balance. ACL is responsible for the diagnosis of joint position, kinesthesia, and equilibrium. Equilibrium is a complex interaction between sensory perceptions and motor responses [32]. Most likely, the reason for improving functional outcomes in the Wii group should be attributed to decreasing the pain as well as improving balance. The most outstanding finding in the present study was the positive impact of ES-based exergames on the improvement of static and dynamic balance.

Our findings revealed a significant improvement in the stability scores in the static BESS test in the Wii group. Karakoc et al. have gained no significant difference in the postural sways between the control and virtual rehabilitation group [15]. In agreement with the current study, Wibelinger et al. demonstrated a remarkable improvement in equilibrium parameters in the Wii group, though, in contrast to the present study, their patients were elderly individuals with knee osteoarthritis [28]. Moreover, in a case of rehabilitation with the Wii Fit following after PCL reconstruction, Puh et al. showed a reduction in the number of postural sways and an improvement in open and closed eye balance outcomes [29]. According to the favorable results of this research, the utilization of ES-based exergames could stimulate the proprioceptive receptors in reconstructed ACL and hence increase proprioceptive function and static postural stability.

This study applied the modified SEBT test in the anterior, posteromedial, and posterolateral directions to evaluate the dynamic balance. Significant differences exist between the groups in all directions of the modified SEBT test. So far, no scientific evidence supports the positive effect of exergames with Wii Fit on dynamic balance, especially after ACLR. However, based on our findings, it seems that exercise-therapy with Wii Fit using ES is an effective approach to improve the dynamic stability in ACL-reconstructed athletes.

The finding of the present study also showed that the Wii Fit exergaming was very pleasant for the participants in the Wii group, and the patients' motivation to exercise was much higher and they participate eagerly in rehabilitation programs which were very encouraging for the researchers, besides, no dissatisfaction or side effects were reported by the patients. Wii Fit exergames therapy with an ES exclusively for use in rehabilitation clinics can improve patients' function, save time and resources. Besides, during COVID-19 pandemics, patients can receive their rehabilitation program by the ES and do it alone via Wii Fit.

## Limitations

Although our study investigated the effectiveness of ES-based Wii Fit protocols compared to conventional rehabilitation outcomes in patients following ACLR, it is important to acknowledge the limitations of our study. One such limitation is the small number of patients and the lack of follow-up due to the COVID-19 pandemic. Additionally, our study was conducted in a controlled environment, which may not accurately reflect real-world conditions where patients may face different challenges and experiences during their rehabilitation process.

Another limitation of our study is that the duration was limited, and we were unable to assess the long-term effects of the ES-based Wii Fit protocols. While our research suggests that ES-based Wii Fit protocols may be a promising alternative to conventional rehabilitation outcomes, it is necessary to conduct research with larger sample sizes and longer durations to fully understand the potential benefits of this therapy.

Furthermore, we did not use digital systems for balance assessment to evaluate postural stability parameters, which may have limited the accuracy of our findings. Also, scientific evidence is lacking to support ES for rehabilitation after ACLR, especially for neuromuscular training.

## Conclusion

The findings support the feasibility of the application of ES-based exergames with the Wii Fit system in the rehabilitation of ACLR as a safe and well-tolerated approach. Our results showed that ES-based exergames was associated with a greater improvement of function and balance. The findings of the study, also, demonstrate that the exergames were effective in significant progress of the ACL-reconstructed athletes' clinical outcomes following a 12-supervised session.

## Data Availability

All data relevant to the study are included in the article. The datasets generated during and/or analyzed during the current study are available from the corresponding author, Arezoo Abasi, upon reasonable request. Please contact Arezoo Abasi at arezoo_abasi@modares.ac.ir for data requests. The data will be provided in a de-identified format and subject to any necessary ethical and legal approvals. Any materials used in this study are available upon request from the corresponding author.
